# A Review of Nanotechnology in microRNA Detection and Drug Delivery

**DOI:** 10.3390/cells13151277

**Published:** 2024-07-30

**Authors:** Hsiuying Wang

**Affiliations:** Institute of Statistics, National Yang Ming Chiao Tung University, Hsinchu 300093, Taiwan; wang@stat.nycu.edu.tw

**Keywords:** drug delivery, nanotechnology, microRNA, microRNA detection, sensor

## Abstract

MicroRNAs (miRNAs) are small, non-coding RNAs that play a crucial role in regulating gene expression. Dysfunction in miRNAs can lead to various diseases, including cancers, neurological disorders, and cardiovascular conditions. To date, approximately 2000 miRNAs have been identified in humans. These small molecules have shown promise as disease biomarkers and potential therapeutic targets. Therefore, identifying miRNA biomarkers for diseases and developing effective miRNA drug delivery systems are essential. Nanotechnology offers promising new approaches to addressing scientific and medical challenges. Traditional miRNA detection methods include next-generation sequencing, microarrays, Northern blotting, and reverse transcription-quantitative polymerase chain reaction (RT-qPCR). Nanotechnology can serve as an effective alternative to Northern blotting and RT-qPCR for miRNA detection. Moreover, nanomaterials exhibit unique properties that differ from larger counterparts, enabling miRNA therapeutics to more effectively enter target cells, reduce degradation in the bloodstream, and be released in specific tissues or cells. This paper reviews the application of nanotechnology in miRNA detection and drug delivery systems. Given that miRNA therapeutics are still in the developing stages, nanotechnology holds great promise for accelerating miRNA therapeutics development.

## 1. Introduction

Nanotechnology, a field pertaining to the utilization of technology at the nanoscale with practical real-world applications, has rapidly expanded since the late 20th century [1]. This technology has quickly infiltrated every realm of science, providing substantial alternative approaches to addressing scientific and medical inquiries and challenges [2]. 

Materials at the nanoscale exhibit unique properties distinct from larger counterparts, unlocking new avenues for biological research [3]. Nanoparticles, due to their small size, customizable surfaces, enhanced solubility, and versatility, offer biologists unprecedented opportunities for exploration. Over the last decade, there has been rapid progress in the development of mobile micro- and nanorobots, which can execute various functions within the biomedical domain, including targeted drug delivery, localized biopsy, bioanalysis, cell sorting, detoxification, and isolation of targets [4]. Nanotechnology enhances tissue regeneration through targeted drug delivery, nanoscale biomaterials, and advanced culture systems, revolutionizing regenerative medicine [5].

microRNA (miRNA) is a small non-coding RNA, about 21–24 nucleotides long, that plays key roles in cell differentiation, development, cell cycle regulation, and apoptosis [6,7]. miRNAs can regulate up to 30% of protein-coding genes in the human genome and are known to be involved in many diseases [8]. In cancer cells, miRNAs often become dysregulated due to mechanisms such as abnormal transcriptional control, epigenetic changes, and defects in miRNA biogenesis [9,10]. In addition, circulating miRNAs exhibit abnormal expression in various other diseases, including neurological diseases and aging-related conditions [11,12]. miRNAs have gained considerable interest in diagnostics and therapy of Alzheimer’s disease, Parkinson’s disease, and amyotrophic lateral sclerosis [13]. miRNAs are crucial for skeletal muscle development, homeostasis, and aging. They have been shown to regulate molecules and pathways involved in muscle aging and rejuvenation, making them potential biomarkers and intervention targets for sarcopenia [14]. A serum miRNA panel was developed to identify patients with all stages of gastric cancer from a high-risk population [15]. Serum exosomal miRNAs have been reported as robust biomarkers for detecting gastric cancer [16]. miRNAs can also serve as valuable biomarkers for encephalitis, depression, and diabetes mellitus [17,18,19].

Different techniques such as next-generation sequencing, microarray, Northern blot, and reverse transcription-quantitative polymerase chain reaction (RT-qPCR) are commonly employed to detect miRNAs [20]. The microarray and sequencing methods are both conventional high-throughput analyses for exploring miRNA biomarkers [21]. The miRNA identified by the microarray and sequencing methods can be validated by the Northern blot and RT-qPCR methods. The Northern blot method comes with certain drawbacks, including high expenses and limited specificity, and RT-qPCR, though highly specific, can be expensive when dealing with large numbers of samples or targets. Nano-based electrochemical techniques have the potential to be used as innovative methods for identifying different miRNAs [22]. These techniques are emerging technologies with variable costs, and hold promise for cost-effective, high-sensitivity miRNA detection. 

In addition to nano-based electrochemical techniques that will be reviewed in this study, DNA nanotechnology shows great promise for the development of innovative miRNA biosensors. Nanomaterials can be constructed using two main approaches: top-down and bottom-up [23]. The top-down approach involves starting with a bulk material and breaking it down to nanoscale sizes, such as with lithography. The bottom-up approach involves assembling nanoscale materials from smaller building blocks, like macromolecules. DNA, for example, can be used in bottom-up construction to create nanostructures. Given that nucleic acids naturally act as biosensors for miRNAs, miRNA detection has been a gateway to biosensing applications in DNA nanotechnology. The field of DNA nanotechnology holds significant potential for developing innovative miRNA biosensors, which are useful for lab-based biosensing and could play a crucial role in disease diagnostics [24]. 

Non-coding RNAs (ncRNA), including miRNAs, can selectively silence specific genes, enabling them to target disease-related proteins. This capability has garnered significant interest from biotech and pharmaceutical companies, leading to the approval of the first ncRNA therapeutic by the FDA in 2018 [25]. Epigenetic regulation by miRNAs has shown significant therapeutic potential in controlling genetic activity across various cancers [26]. As a result, therapeutic approaches involving miRNA mimics and anti-miRNAs, which can either boost miRNA expression or reduce the levels of abnormally expressed miRNAs, are highly desired for effectively controlling miRNA levels [27]. Therefore, carrier vehicles that facilitate efficient and safe delivery of miRNA-based therapeutics are essential for their success in clinical applications. 

The exploration of the utility and potential of nanotechnology in miRNA formulation and delivery has opened up promising avenues in the field of biomedicine [28]. Nanotechnology can improve the stability, specificity, and delivery efficiency of miRNA drugs. Utilizing nanocarriers allows miRNA therapeutics to more effectively penetrate target cells, minimize degradation in the bloodstream, and be released in designated tissues or cells. These benefits position nanotechnology as a promising approach for enhancing the efficacy of miRNA therapies and advancing their clinical use. This paper reviews the nanotechnology applications for miRNA detection and miRNA drug delivery.

## 2. MicroRNA

The first miRNA was discovered by Victor Ambros and colleagues in 1993 while studying the development of the nematode *Caenorhabditis elegans* [29]. This discovery has since opened up a new field of research focusing on the regulatory roles of miRNAs in gene expression. So far, there have been around 2000 miRNAs identified in humans, and it is believed that together they regulate approximately one-third of the genes in the genome [30]. Several miRNA databases, including miRBase and MirGeneDB, have been established. miRBase is a comprehensive online database that serves as a repository for miRNA sequences and annotations [31]. MirGeneDB is another essential database dedicated to the annotation and classification of miRNA genes, which focuses on providing a high-quality, manually curated set of miRNA gene annotations [32]. 

The most promising role of miRNAs may be as potential biomarkers for diseases [33]. The abnormal expression of miRNAs has been linked to a range of human diseases such as cancers/tumors, diabetes, viral infections, cardiovascular diseases, neurodegenerative diseases, and other diseases [34]. miRNAs play a role in the onset and advancement of cancers, with their expression serving as a significant prognostic tool for cancer [10]. The aberrant expression of miRNAs in the blood is linked to both type 1 diabetes mellitus and type 2 diabetes mellitus, sometimes detectable several years before the disease becomes apparent [35]. miRNAs have not only been discovered to be useful disease biomarkers but can also be used to explore the association between diseases. The link between depression and gastroesophageal reflux has been explored based on the phylogenetic analysis of their miRNA biomarkers [36]. The association between migraine and depression has been examined based on miRNA biomarkers and cohort studies [37]. In addition, the distribution of the pairwise distance between human miRNAs has been established, which may be used to explore the association between diseases [38,39]. 

Since miRNA biomarkers are useful tools for disease diagnosis and prognosis, it is important to accurately identify miRNA biomarkers for various diseases. To study the miRNA biomarkers, the expression levels of miRNAs in the bodily fluids or tissues of patients can be compared with those of control subjects to identify miRNA biomarkers. There are several methods for detecting miRNAs in fluids or tissues, including next-generation sequencing, microarray, Northern blot, and RT-qPCR [40,41]. The microarray and sequencing methods are both conventional high-throughput analyses used to explore miRNA biomarkers. miRNAs identified through these methods can be further validated using the Northern blot and RT-qPCR techniques.

miRNA microarray technology relies on nucleic acid hybridization between target molecules and their complementary probes [42]. Isolated miRNAs are labeled with a fluorescent dye and then hybridized to the miRNA microarray, leading to the specific binding of labeled miRNAs to corresponding probes. This method allows for the high-throughput analysis of thousands of miRNAs simultaneously. Next-generation sequencing for miRNA detection involves converting miRNAs into cDNA, followed by adapter ligation and polymerase chain reaction (PCR) amplification [43,44]. Microarray and sequencing methods have been compared in the literature [45,46].

Northern analysis is a commonly used method for miRNA analysis because it is typically accessible to laboratories and does not require specialized equipment or advanced technical expertise [47]. However, traditional DNA oligonucleotide probes used in Northern blot technology have significant drawbacks, including low sensitivity and being time-consuming. Other improved Northern blotting techniques have been proposed. miRNA detection by Northern blotting using locked nucleic acid probes was developed [48,49]. Locked nucleic acid-modified oligonucleotide probes were used to enhance the efficiency of hybridization in Northern blotting [50]. 

PCR is a widely used technique in molecular biology to amplify a specific segment of DNA, generating millions to billions of copies of a particular DNA sequence from a small initial sample. Currently, PCR is among the most invaluable techniques utilized in bioscience, diagnostics, and forensic science [51]. However, miRNAs pose a challenge for PCR amplification due to the stable hairpin structure of the miRNA precursor and the fact that the mature miRNA is similar in size to a standard PCR primer [52]. Therefore, improved PCR is often used to detect miRNAs. Quantitative PCR (qPCR), also known as real-time PCR or quantitative real-time PCR, is a PCR-based method that links the amplification of a specific DNA sequence with the measurement of the concentration of that DNA in the reaction [53]. The real-time reverse transcription polymerase chain reaction (RT-PCR) employs fluorescent reporter molecules to track the generation of amplification products throughout each PCR cycle [54]. RT-qPCR is a technique that combines RT-PCR with qPCR to measure RNA levels by using cDNA in a qPCR reaction, enabling the rapid detection of gene expression changes [55]. 

In addition to these methods, nano-based electrochemical techniques have the potential to be used as innovative detection methods for identifying different miRNAs [22]. Nanomaterials-based fluorimetric techniques offer the potential for highly sensitive, efficient, and selective detection of miRNAs [56]. 

Moreover, compared with other small molecules, such as siRNA, the progress of miRNA therapeutics is relatively slow. Although RNAi (RNA interference) therapeutics have been on the rise, with the FDA approving two siRNA-based drugs, Patisiran in 2018 and Givosiran in 2019, there has been less news about the progress of miRNA drugs compared with siRNA [57]. Many miRNA drugs are under development. miRNA-derivative clinical nucleotide drugs have proven effective in early-stage trials for conditions like malignant pleural mesothelioma and hepatitis C infection, with many currently in clinical trials [58]. One of the obstacles to the progress of miRNA therapeutics is the challenge of efficiently developing miRNA drug delivery systems. Nanomaterials are being utilized for the delivery, release, and treatment of miRNA. They enhance the stability, bioavailability, and tissue specificity of miRNAs while minimizing effects on non-target tissues.

## 3. Nanotechnology in microRNA Detection

Nano-based electrochemical techniques for miRNA detection may be categorized into methods using inorganic nanoparticles, carbon-based nanoparticles, and nanopore technology.

### 3.1. Inorganic Nanoparticles

#### 3.1.1. Magnetic Nanoparticles

Nanoparticle-based biosensors, particularly magnetic nanoparticles (MNPs), have superior efficiency in detecting miRNAs from body fluids compared to traditional methods [59]. miRNAs in urine are promising biomarkers for early disease diagnosis. However, conventional extraction methods face challenges due to low concentrations and diverse carriers of miRNAs. MNPs with carboxylic acid coating have been shown to effectively isolate low-concentration miRNAs from dilute solutions such as urine and cell culture medium by adsorbing proteins and forming a protein corona, facilitating easy aggregation and precipitation [60]. MNPs offer advantages like biocompatibility and high stability that enable sensitive and specific detection of miRNAs from complex samples like urine and blood [61].

There is significant interest in measuring miRNA levels in blood. The challenge lies in developing methods to monitor miRNA expression across wide concentration ranges and in minute quantities directly in patient blood. A novel sensor, utilizing electric-field-induced reconfiguration of gold-coated MNPs with probe DNA (DNA–Au@MNPs), demonstrates high sensitivity for direct miRNA analysis in whole blood [62]. 

A sensor employing two distinct magnetic nanoprobes (DNA1/Fe3O4 NPs/Thi and DNA2/Fe3O4 NPs/Fc) with a target-triggered hybridization chain reaction strategy was developed for simultaneous detection of miR-141 and miR-21 [63]. Multiple miRNAs could be simultaneously assessed with high sensitivity, even within cell lysates. Iron oxide nanoparticles have garnered significant attention due to their unique properties, including superparamagnetism, a high surface-to-volume ratio, increased surface area, and ease of separation [64]. An ultrasensitive and specific electrochemical sensor utilizing graphene-oxide-loaded iron oxide (GO/IO hybrid material) has been developed for detecting cancer-related miRNA [65].

#### 3.1.2. Gold Nanoparticles

Gold nanoparticles (GNPs) have been utilized in biomedicine for centuries. Their unique electronic properties and colors have gained significant attention, extending beyond ancient medical and artistic applications [66]. Nanogold has superiority over other nanomaterials due to optimized production methods that yield diverse sizes and shapes with unique properties [67]. 

GNPs can be employed as probes for the detection of miRNAs. A surface for miRNA detection is developed using a composite of probe DNA, GNP, and dendrimers, coupled with enzymatic amplification. It shows promise for early cancer diagnosis, with high sensitivity [68]. A miRNA biosensor integrates surface-enhanced Raman spectroscopy and electrochemical techniques, compensating for each other’s weaknesses [69]. This approach, utilizing GNP, enables specific detection of miR-155 with a wide dynamic range in human serum. 

In addition, GNP-based sensors for miRNA detection can be classified into two different categories: solution-based sensors and solid support-based sensors [70]. The former encompasses sensing platforms where GNPs are dispersed within a solution, such as colorimetric and fluorescent sensors. In the latter category, GNPs are either combined with or affixed to a solid support, as seen in electrochemical sensors, surface plasmon resonance sensors, lateral flow strip sensors, and glass-slide-based sensors.

#### 3.1.3. Quantum Dots

Photoluminescent semiconductor nanoparticles, known as quantum dots (QDs), have garnered significant attention from materials scientists, physicists, chemists, and biologists worldwide [71]. Their high quantum yield, adjustable emission spectrum, and outstanding photostability make them particularly appealing [72]. These distinctive optical properties make QDs highly promising for various biomedical applications. 

The relatively small size and narrow size distribution of QDs are especially important for miRNA assays. miRNAs are small analytes and single-stranded oligonucleotide sequences that can be used as components in QD–DNA conjugates [73]. By integrating QD-based biosensors with amplification techniques, it becomes possible to detect target miRNA at a single-particle level, reaching detection limits at the attomolar scale [74]. In addition, diagnosing diseases through the detection of individual miRNAs may result in a high false-positive rate. As a result, a fluorescence biosensor method for the one-step simultaneous detection of multiple miRNAs has been developed, employing single-stranded DNA functionalized double QDs and black hole quencher-decorated magnetic nanobeads [75].

miRNAs serve as valuable biomarkers for cancer diagnosis. The application of QDs as sensing probes for detecting cancer-related miRNAs and other biomarkers has been extensively studied [76]. The fluorescence properties of biocompatible nitrogen-doped graphene QDs synthesized via a bottom-up approach from a single glucosamine precursor were utilized to detect a specific pancreatic-cancer-derived miRNA (pre-miR-132) [77]. A two-color water-soluble QD system has been developed for miR-155 detection, and this assay was applied for the determination of miR-155 in breast cancer cells [78].

QDs prepared in the organic phase typically exhibit high photoluminescence quantum yield, narrow photoluminescence half-width, good monodispersity, and high stability. However, they also have several disadvantages, including the use of toxic reagents, high experimental costs, and increased safety concerns during the synthesis process [79]. Certain semiconductor QDs contain hazardous substances such as cadmium, arsenic, selenium, and mercury, which pose significant disadvantages such as cytotoxicity. Consequently, these QDs may be neither environmentally friendly nor biodegradable [80].

### 3.2. Carbon-Based Nanoparticles

Cancer diagnosis and treatment are hindered by drug-resistant tumor cells. Nanotechnology, particularly carbon-based theranostic nanoparticles, provides promising solutions due to their unique properties and applications in tissue engineering, drug delivery, imaging, and biosensors [81]. Carbon nanomaterials include graphene, graphene oxide and reduced graphene oxide, carbon nanotubes, carbon dots, diamond, fullerene, carbon nanofibers, carbon nanohorns, nanoporous carbon, nano carbon black, and their composites [82]. One of the attractive applications of carbon-based nanomaterials is in biosensors. The use of various nanomaterials in the development of electrochemical biosensors has significantly enhanced their detection sensitivity. Notably, carbon nanomaterials have garnered increasing attention due to their unique electrical, optical, physical, and chemical properties. These materials offer a large surface area, excellent biocompatibility, and high conductivity, making them particularly valuable in this field [83]. 

Carbon nanodots have been identified as a strong candidate to replace the highly hazardous metallic semiconductor class of QDs [80]. A simple carbon-nanodot-based electrogenerated chemiluminescence biosensor was successfully used for the sensitive and selective detection of miRNA-21 directly in serum samples from heart failure patients without the need for prior RNA extraction or amplification processes [84]. A carbon-based DNA framework nano–bio interface using DNA tetrahedrons on screen-printed carbon electrodes (SPCE) improves biosensing for medical diagnostics, food safety, and environmental monitoring by enhancing signal-to-noise ratio and sensitivity [85]. The improved SPCE could be applied for the detection of a variety of bioactive molecules, including miRNA-141. 

Devices based on graphene and its derivatives have shown great potential as miRNA sensors because of their remarkable electrical, chemical, optical, mechanical, and structural properties [86]. A highly sensitive electrochemical biosensor for detecting miRNA was created using tungsten-oxide–graphene composites, combined with catalyzed hairpin assembly target recycling and enzyme signal amplification [87]. An exceptionally sensitive electrochemical biosensor has been developed to detect miRNA, incorporating magnesium oxide nanoflowers and hybrids of graphene oxide and gold nanoparticles, in combination with an electrochemical–chemical–chemical detection system [88]. 

### 3.3. Nanopore Technology

Nanopore technology is a cutting-edge technique used in the fields of life sciences and biotechnology. It involves the use of nanometer-scale pores, known as nanopores, to analyze and sequence molecules at a single-molecule level. By incorporating a nanoscale hole in a thin membrane and measuring the electrochemical signals, this technology enables the investigation of nucleic acids and other biomacromolecules [89]. Nanopores have applications in molecular sensing and sequencing, chemical catalysis, and biophysical characterization [90].

A system that utilizes DNA computing and nanopore decoding for recognizing miRNA expression patterns has been developed [91]. The encoded information within miRNA, which is hybridized to HP-dgDNA, is deciphered through nanopore sensing. This decoding occurs through analysis of the unzipping time of the hybridized strands as the diagnostic construct moves through the nanopore. A platform was used for the rapid electronic detection of probe-hybridized miRNAs from cellular RNA [92]. In this platform, a target miRNA is first hybridized to a probe. The resulting probe duplex is then enriched through binding to the viral protein p19. Finally, the abundance of the duplex is quantified using a nanopore.

Nanopore sequencing was conducted using DNA-barcoded molecular probes engineered to recognize a variety of analytes [93]. This technique enables highly multiplexed and simultaneous quantitative detection of many targets, including miRNAs, proteins, and neurotransmitters, by analyzing the translocation dynamics of each probe as it passes through a nanopore.

**Figure 1:**
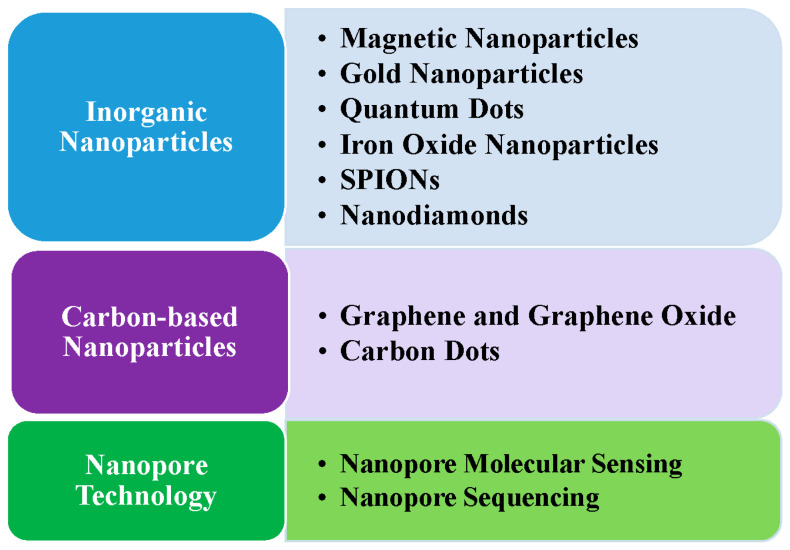
The nanomaterials used in miRNA detection.

Figure 1 summarizes the nanomaterials for miRNA detection reviewed in this paper.

In addition, Table 1 provides several advantages and disadvantages of these nanotechnologies for miRNA detection that are discussed in this section. 

## 4. Drug Delivery

Nanocarriers are widely used in the delivery, release, and treatment of miRNA. These carriers can improve the stability, bioavailability, and tissue specificity of miRNAs while reducing the impact on non-target tissues. There are several common nanocarriers used for miRNA applications, including inorganic nanoparticles, polymeric nanoparticles, lipid nanoparticles, and carbon-based nanomaterials. 

### 4.1. Inorganic Nanoparticles

Inorganic nanoparticles, such as gold and iron oxide, typically have good stability and biocompatibility and can achieve targeted delivery and release of miRNA through surface modification. Inorganic nanoparticles have garnered significant attention as potential diagnostic and therapeutic tools in oncology [94]. 

The utilization of GNPs as carriers for diverse molecules, such as miRNA/siRNA, is driven by their distinctive physical and chemical attributes, such as optical and electrical properties, setting them apart from commonly employed polymeric or lipophilic alternatives [95]. Regulating the osteogenic differentiation of bone mesenchymal stromal cells (MSCs) is crucial for bone regeneration. To facilitate this, surface-engineered, ultra-small GNPs were developed as highly efficient miR-5106 delivery systems to control MSC differentiation [96]. Emerging gene-silencing approaches utilizing miRNAs aim to enhance the therapeutic capabilities of human MSCs. MNP-mediated transfection has demonstrated long-term efficacy, offering a beneficial method for successful genetic modification of stem cells [97]. In addition, multifunctional nanoplatforms were employed for miRNA-101 delivery and near-infrared (NIR) thermal therapy to trigger apoptosis in breast cancer cells [98]. Gold nanorods or nanospheres coated with graphene oxide were prepared and functionalized with polyethylene glycol as a stabilizer and poly-L-arginine (P-L-Arg) as a targeting agent. 

Silica is widely found in nature and is used for various purposes. Diatoms are single-celled algae that are known for their unique and intricate cell walls made primarily of silica [99]. Multifunctional core-shell mesoporous silica nanoparticles, sized between 60 and 160 nm, were engineered as delivery agents for antitumoral miRNA [100]. These versatile nanoparticles demonstrated enhanced uptake into epidermal growth factor receptor (EGFR)-overexpressing T24 bladder cancer cells via receptor-mediated cellular internalization. Combination therapies incorporating siRNA and miRNA hold tremendous promise in cancer treatment. However, the clinical application of siRNA/miRNA combinations is hindered by the limited availability of safe and efficient systemic delivery systems with adequate tumor-penetrating and endosomal escaping capabilities. To address this challenge, multifunctional tumor-penetrating mesoporous silica nanoparticles capable of simultaneously delivering siRNA (siPlk1) and miRNA (miR-200c) have been developed [101]. 

Calcium carbonate (CaCO_3_) is a widely studied material in bioinspired mineralization, given its abundance in various organisms such as oyster shells, corals, ivory, enamel, and bone [102]. CaCO_3_ nanoparticles have been investigated in miRNA delivery [103]. miR-375 and sorafenib were co-loaded into lipid-coated calcium carbonate nanoparticles to treat hepatocellular carcinoma (HCC) [104]. These nanoparticles could facilitate the delivery of sorafenib and miR-375 to HCC cells and tumor tissues, enhancing drug retention time within the tumor. Using a CaCO_3_-based delivery system, pDNA miR-200c has been shown to effectively increase miR-200c expression in oral squamous cell carcinoma cells and suppress oncogenic activities in a preclinical animal model [105].

In addition, the synthesis of layered gadolinium hydroxychloride (LGdH) nanoparticles as a miRNA therapy delivery platform has been reported to inhibit mature miR-10b in metastatic breast cancer cell lines [106]. LGdH nanoparticles exhibit a good cellular uptake profile, resulting in the inhibition of miR-10b function in vitro.

In recent years, mild hyperthermia (40–45 °C), especially magnetic hyperthermia, has been increasingly investigated as an adjuvant that can effectively sensitize tumors to chemotherapy and radiotherapy as well as induce apoptosis. Highly magnetic zinc-doped iron oxide nanoparticles (ZnFe2O4) can be used to deliver a miRNA that targets multiple downstream pathways modulated by heat shock proteins and induces magnetic hyperthermia to enhance the treatment of cancer cells [107]. In addition, superparamagnetic iron-oxide-based nanoparticles (SPIONs) are inorganic nanomaterials with magnetic components and diameters ranging from 10 to 100 nm [108]. SPIONs could be utilized for the delivery of miRNA therapeutics and may provide highly targeted therapy for cancer patients. 

### 4.2. Polymeric Nanoparticles

Versatile protamine nanocapsules were used to restore miR-145 levels as an anticancer therapy, which can interfere with tumor growth in colorectal cancer cells [109]. miR-146a was effectively adsorbed onto poly(glycerol adipate-co-ω-pentadecalactone) (PGA-co-PDL) nanoparticles to target and reduce IRAK1 gene expression for the treatment of chronic obstructive pulmonary disease [110]. A surface-functionalized nanoparticle delivery system using poly(D,L-lactide-co-glycolide)/poly(L-lactide)-block-poly(ethylene glycol)-folate (PLGA/PLA-PEG-FA) was developed to load miR-204-5p (FA-NPs-miR-204) [111]. The therapeutic efficacy of FA-NPs-miR-204 was tested in the Luc-HT-29 xenograft tumor model in vivo. The results showed that this miRNA delivery system could target tumor tissue and exert tumor-suppressive function. Poly (lactic-co-glycolic acid) (PLGA)-based nanoparticles delivered phosphorothioate and peptide-nucleic-acid-based anti-miRs-141-3p for potential stroke therapy was studied [112]. The results showed that nanoparticles encapsulating anti-miRs-141-3p probes could be used as a potential treatment for ischemic stroke.

Bioreducible poly(β-amino ester) nanoparticles were developed to achieve high intracellular delivery efficacy, low cytotoxicity, endosomal escape, and promotion of cytosol-targeted, environmentally triggered cargo release for miRNA delivery to tumor-propagating human cancer stem cells [113]. These nano-miRs efficiently deliver single and combined miRNA mimics into human glioblastoma cells in vitro, inhibiting their stem cell phenotype.

Chitosan nanoparticles (chNPs) were engineered to deliver functional miRNA mimics to macrophages [114]. Their impact on reverse cholesterol transport was investigated in vivo. Utilizing a cross-linked chitosan polysaccharide polymer, these nanoparticles can effectively shield and transport exogenous miR-33 to naïve macrophages. 

### 4.3. Lipid Nanoparticles

Solid lipid nanoparticles are nanocarriers consisting of a lipid core surrounded by a monolayer surfactant shell, which stabilizes them and enables the formation of aqueous dispersions at the nanometer scale [115]. Lipid nanoparticles can enter cells through endocytosis and release miRNA to regulate gene expression. Lipid miRNA delivery systems may be the most extensively studied non-viral carriers due to their biocompatibility, biodegradability, ease of production, low toxicity and immunogenicity, and the ability to easily modify them for targeted delivery strategies [116].

The utilization of liposomes as a carrier for drug delivery in treating diverse diseases, notably cancer, is experiencing rapid growth. miRNA can be encapsulated within liposomes of nanometer size and preserved in a dried state for months, and retain their encapsulation even after prolonged periods of storage [117].

miR-122, a liver-specific tumor-suppressor miRNA, is often downregulated in HCC. A cationic lipid nanoparticle formulation, LNP-DP1, was developed to deliver miR-122 to HCC cells, aiming to restore normal gene expression [118]. The miR-122 mimic in LNP-DP1 worked effectively in HCC cells without causing widespread toxicity. When injected directly into tumors, LNP-DP1 with miR-122 mimic suppressed HCC xenograft growth by about 50% within 30 days, correlating with reduced target gene activity and decreased angiogenesis.

### 4.4. Carbon-Based Nanomaterials

Carbon nanomaterials and their unique physical and chemical properties, such as their capability to protect DNA, have attracted significant attention in the field of nanomedicine and as non-viral carriers for therapeutic genes [119]. These materials include zero-dimension fullerenes and carbon dots, one-dimensional carbon nanotubes (CNTs), and two-dimensional graphene [120].

A fullerene nanospherical miRNA was developed to overcome resistant breast cancer [121]. This chemically synthesized fullerene nanospherical miRNA can deliver miR-27b into resistant cancer cells, escape from lysosomes, and release miR-27b into the cytoplasm. Carbon dots (Cdots) were utilized to deliver locked-nucleic-acid (LNA)-based suppressors, resulting in the specific inhibition of viral miRNAs [122]. This inhibition effectively suppresses the proliferation of Kaposi’s sarcoma-associated herpesvirus (KSHV)-associated primary effusion lymphoma (PEL) cells. Specifically, a combination of Cdots and LNAs reduces the levels of KSHV miR-K12-1, miR-K12-4, and miR-K12-11, inducing apoptosis and impeding PEL cell proliferation. These results showcase the feasibility of employing Cdots for delivering miRNA suppressors to target viral cancers. 

Nanodiamonds (NDs) are a distinctive type of nanoparticle within the carbon nanomaterials group, distinguished by their outstanding biocompatibility and inherent physicochemical properties [123]. NDs are innovative nanocrystalline carbon particles capable of delivering miRNAs. The potential of Raman spectroscopy and the fluorescence properties of NDs have been investigated for applications in bioimaging and drug delivery tracking [124]. A nanodiamond-based system was developed to deliver miR-34a to breast cancer tissues, where NDs were conjugated with miR-34a using the chemical crosslinker polyethyleneimine [125]. 

Polymer-functionalized CNTs were developed to deliver miRNAs to endothelial cells for the regulation of angiogenesis [126]. The CNTs were coated with two different polymers, polyethyleneimine or polyamidoamine dendrimer, and subsequently conjugated with miR-503 oligonucleotides. Both polymer-coated CNTs exhibited reduced toxicity compared to pristine CNTs or the polymers alone.

Carbon-based quantum particles, notably spherical carbon quantum dots (CQDs) and nanosheets such as graphene quantum dots (GQDs), are an emerging class of quantum dots distinguished by their distinctive properties arising from the quantum confinement effect [127]. A multifunctional nanocomposite comprising poly(l-lactide) (PLA) and polyethylene glycol (PEG)-modified GQDs (f-GQDs) was suggested for the concurrent imaging of intracellular miRNA and the joint delivery of genes to improve therapeutic effectiveness [128]. GQDs–miRNA-223 linked by disulfide bonds are grafted onto the monocyte membrane via a carefully designed C18-peptide (C18P) that contains a hydrophobic end to afford the designed monocyte–C18P–GQDs–miR-223 architecture. The system can reach and enter the interior of the plaque and release the GQDs–miRNA via C18P digestion. A monocyte surface-engineered gene-delivery system based on GQDs has been developed [129]. The connection between GQDs and miR-223 is achieved through disulfide bonds, and they are attached to the monocyte membrane using a precisely engineered C18P that includes a hydrophobic end. This process results in the creation of the designed monocyte–C18P–GQDs–miR-223 structure. This system could effectively penetrate and enter the interior of the plaque, allowing for the release of GQDs–miRNA upon digestion of C18P.

**Figure 2:**
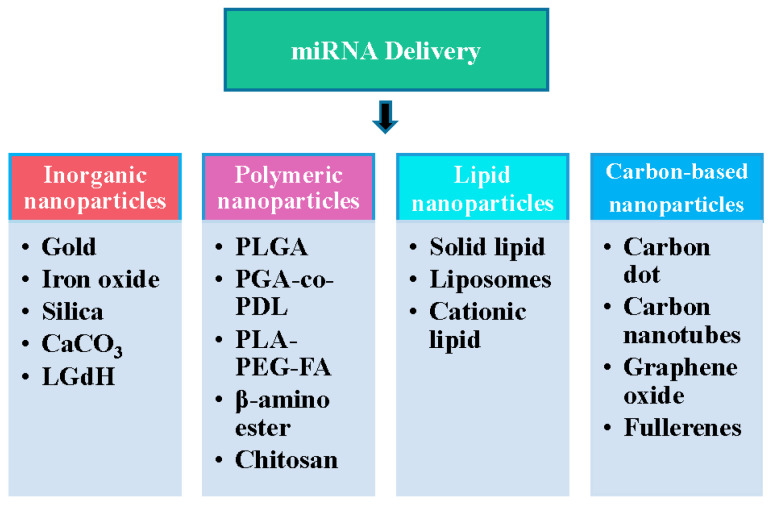
The nanomaterials used in miRNA drug delivery.

Figure 2 summarizes the nanomaterials for miRNA delivery reviewed in this paper. 

## 5. Discussion

miRNAs are promising as disease biomarkers and therapeutic agents. miRNA treatment involves utilizing miRNA molecules as therapeutic agents to regulate gene expression and potentially treat various diseases. miRNA-based therapeutics can include miRNA mimics, which restore the function of downregulated miRNAs, or antimiRs, which inhibit overexpressed miRNAs [130]. AntimiRs are single-stranded oligonucleotides that bind to complementary sequences of miRNAs, disrupting their activity and/or processing. This leads to increased expression of target tumor-suppressor genes. In contrast, miRNA mimics play an opposing role by upregulating miRNA expression, consequently downregulating the expression of target genes, such as oncogenes [131].

Restoring the function of diminished tumor-suppressor miRNAs to normal levels using miRNA mimics is a promising cancer treatment strategy [132]. These can boost the activity of natural miRNAs and revive the expression of miRNAs that suppress tumors. Restoring the miRNA let-7 has been demonstrated to significantly inhibit tumor growth in mouse models [133]. Inhibition of miRs by antimiRs might be a therapeutic option for many diseases, but systemic inhibition can have adverse effects. Light-activatable antimiRs could efficiently and locally restrict target miR activity in vivo. An antimiR-92a was used to establish a therapeutic benefit in diabetic wound healing [134]. AntimiR-92a is modified with photolabile protecting groups, known as ‘cages’. Upon irradiation, these caged antimiR-92a molecules are activated when injected intradermally, without significantly impacting miR-92a expression in other organs. The light activation of caged antimiR-92a enhances healing in diabetic mice to a degree comparable to that of conventional antimiRs.

miRNAs have an important function: cross-kingdom regulation, a phenomenon where miRNAs from one species can regulate gene expression in another species [135]. Exogenous plant miRNAs are found in the sera and tissues of various animals, indicating that these miRNAs are primarily acquired orally through food intake [136]. miRNA-2911, an atypical miRNA encoded by honeysuckle, can directly target a broad spectrum of influenza A viruses (IAVs), and it is the first active component identified in traditional Chinese medicine that directly targets various IAVs [137]. Understanding cross-kingdom miRNA regulation opens up new possibilities for developing miRNA-based therapies for diseases by exploiting natural miRNAs or designing synthetic ones. However, the debate on the possibility of cross-kingdom gene regulation by orally acquired plant miRNAs has been ongoing for nearly a decade without reaching a definitive conclusion [138]. Cross-kingdom gene regulation by orally acquired plant miRNAs may occur under specific conditions, influenced by factors such as the types of plant miRNAs, the delivery mechanism, and their concentrations in the plant [138].

While miRNA holds promise for personalized medicine and targeted therapy, miRNA treatment is still in the early stages of development. The challenges in developing miRNA therapy can be categorized into several factors, such as the complexity of miRNA networks, the identification of therapeutic miRNAs, and delivery systems. miRNAs may regulate multiple target genes, and a single miRNA can influence various biological pathways. The same miRNA can be involved in more than one disease [6,11,18,19]. This complexity makes it difficult to predict and control the effects of miRNA-based therapies. The identification of miRNA biomarkers is crucial for the development of miRNA therapy. Different conventional techniques like Northern blotting and RT-qPCR are employed to detect miRNA. Nano-based electrochemical techniques have the potential to be used as innovative and precise detection methods for identifying different miRNAs [22]. In Section 3, the miRNA detection using nanotechnology including inorganic nanoparticles, carbon-based nanoparticles, and nanopore technology is reviewed. In miRNA drug delivery, conventional systems often lack precise targeting of specific cells or tissues. In contrast, nucleic-acid nano-delivery systems present a promising therapeutic strategy for delivering nucleic acids to regulate gene expression in specific cells [139]. The nanotechnology drug delivery systems used for miRNA applications, including inorganic nanoparticles, polymeric nanoparticles, lipid nanoparticles, and carbon-based nanomaterials, are reviewed in Section 4. Compared with organic nanoparticles, inorganic nanoparticles may have inferior biodegradability properties. The integrity, biodistribution, and fate of several inorganic nanoparticles after in vivo administration have been investigated, and increasing experimental evidence suggests that these compounds may degrade in vivo [140].

The nanomaterials discussed in this study involve various materials sourced from organisms such as chNPs, PLGA, and liposomes. These biomaterials have emerged as outstanding choices for biosensor development [141]. Their unique properties, such as biocompatibility, biodegradability, and ease of modification, make them highly suitable for advancing biomedical applications [142,143,144]. Biopolymeric nanoparticles are increasingly important as nanocarriers for various biomedical applications, facilitating long-term and controlled release at targeted sites [145]. Lipid nanocarriers have been developed as a drug delivery system for oil extracts from Citrus aurantium L. blossoms and Rosa damascene [146]. In addition, there are several bioproduced inorganic particles such as silica and CaCO_3_.

It is important to note that each variant of nanoparticles combined with miRNAs can exhibit unique properties. Each specific case requires its own optimized system. Factors such as cell toxicity, nanoparticle stability, cellular uptake, and cargo release vary significantly across different systems. These differences can determine which system is most useful in particular situations. Therefore, tailored optimization for each application is crucial to maximize the efficacy and safety of nanoparticle-based miRNA delivery systems.

## 6. Conclusions

This paper reviews the use of nanotechnology in miRNA detection and drug delivery. It covers the application of inorganic nanoparticles, carbon-based nanoparticles, and nanopore arrays in miRNA detection, as well as the use of inorganic nanoparticles, polymeric nanoparticles, lipid nanoparticles, and carbon-based nanomaterials in miRNA drug delivery.

While miRNAs hold promise in disease diagnosis and therapy, conventional techniques for miRNA detection and therapy face several drawbacks, including issues with sensitivity, specificity, efficiency, and safety. Nanotechnology can enhance the development of miRNA detection by providing highly sensitive and specific tools that allow for the precise identification and quantification of miRNAs. Through the use of advanced materials such as inorganic nanoparticles, carbon-based nanoparticles, and nanopore arrays, nanotechnology enables the creation of innovative detection platforms that can improve the accuracy, speed, and efficiency of miRNA analysis. These advancements are crucial for early diagnosis and monitoring of various diseases. 

Nanotechnology can enhance the stability, specificity, and delivery efficiency of miRNA drugs. By using nanocarriers, miRNA therapeutics can more effectively enter target cells, reduce degradation in the bloodstream, and be released in specific tissues or cells. These advantages make nanotechnology a promising solution for improving the efficacy of miRNA therapies and advancing their clinical applications.

## Figures and Tables

**Table 1:** Some advantages and disadvantages of these nanotechnologies for miRNA detection reviewed in this paper.

Nanotechnology	Advantage or Disadvantage
Inorganic Nanoparticles	MNPs offer advantages like biocompatibility and high stability that enable sensitive and specific detection of miRNAs. GNPs show promise for early cancer diagnosis with high sensitivity. Iron oxide nanoparticles have unique properties, including superparamagnetism, a high surface-to-volume ratio, increased surface area, and ease of separation. QDs prepared in the organic phase typically exhibit high photoluminescence quantum yield, narrow photoluminescence half-width, good monodispersity, and high stability. QDs have disadvantages, such as cytotoxicity and high cost.
Carbon-Based Nanoparticles	Carbon nanomaterials offer a large surface area, excellent biocompatibility, and high conductivity. Devices based on graphene and its derivatives have remarkable electrical, chemical, optical, mechanical, and structural properties.
Nanopore Technology	Nanopores have applications in molecular sensing and sequencing, chemical catalysis, and biophysical characterization. Nanopore sequencing enables highly multiplexed and simultaneous quantitative detection of many targets.

## Abbreviations

CaCO_3_	calcium carbonate
Cdots	carbon dots
CNTs	carbon nanotubes
CQDs	carbon quantum dots
chNPs	chitosan nanoparticles
C18P	C18-peptide
EGFR	epidermal growth factor receptor
GO/IO	graphene-oxide-loaded iron oxide
GQDs	graphene quantum dots
GNPs	gold nanoparticles
HCC	hepatocellular carcinoma
IAVs	influenza A viruses
LNA	locked nucleic acid
KSHV	Kaposi’s sarcoma-associated herpesvirus
MNPs	magnetic nanoparticles
MSCs	mesenchymal stromal cells
miRNAs	microRNAs
f-GQDs	modified GQDs
NDs	nanodiamonds
ncRNA	non-coding RNAs
NIR	near-infrared
P-L-Arg	poly-L-arginine
PCR	polymerase chain reaction
PLGA	poly (lactic-co-glycolic acid)
PLA	poly(l-lactide)
PEG	polyethylene glycol
PEL	primary effusion lymphoma
qPCR	quantitative PCR
RT-qPCR	quantitative polymerase chain reaction
QDs	quantum dots
RT-PCR	reverse transcription polymerase chain reaction
SPCE	screen-printed carbon electrodes
SPIONs	superparamagnetic iron-oxide-based nanoparticles
